# Robotische Assistenz bei den Aktivitäten des täglichen Lebens am Beispiel der Nahrungsaufnahme

**DOI:** 10.1007/s00391-020-01785-4

**Published:** 2020-10-06

**Authors:** Barbara Klein, Annalies Baumeister

**Affiliations:** Fachbereich Soziale Arbeit & Gesundheit, Frankfurt University of Applied Sciences, Nibelungenplatz 1, 60318 Frankfurt am Main, Deutschland

**Keywords:** Essen, Trinken, Hilfsmittel, Robotik, Unterstützungsbedarf, Eating, Drinking, Assistive technology, Robotics, Need for support

## Abstract

Die Ausübung der Aktivitäten des täglichen Lebens (ADL, auch Basisaktivitäten) ist bei Funktionseinschränkungen und -verlusten, die mit dem Altern, mit Krankheiten oder Behinderung einhergehen, eingeschränkt bzw. nicht mehr möglich. Dafür gibt es heute schon eine Reihe von Hilfsmitteln und sogar robotische Produkte. Ziel dieses Beitrages ist es, exemplarisch für die Basisaktivität Nahrungsaufnahme einen Überblick über die vorhandenen Produkte für Menschen mit einer Tetraplegie aufgrund eines Unfalls oder neurologischer Erkrankungen wie multipler Sklerose oder amyotrophischer Lateralsklerose zu geben. Die Verbreitung dieser Systeme ist heute noch sehr überschaubar. Dazu werden hemmende und fördernde Faktoren für deren Verbreitung und Nutzung aufgezeigt.

## Abstract (en)

Carrying out activities of daily living (ADL, also called basic activities) is limited or even no longer possible due to functional limitations and functional losses associated with aging, (chronic) illness and disabilities. Nowadays, there are a variety of assistive technologies/devices and even robotic products. The aim of this article is to give an exemplary overview of the existing products for the basic activity of food intake for people with tetraplegia caused by accidents or neurological disorders, such as multiple sclerosis or amyotrophic lateral sclerosis (ALS). The dissemination and implementation of these products seems to be relatively low in Germany. Inhibiting and promoting factors for the dissemination and utilization are discussed.

## Ausgangssituation

Die Ausübung der Basisaktivitäten trägt zu einem selbstständigen Leben bei. Für diese Basisaktivitäten, die das Essen und Trinken, Baden/Duschen, An- und Auskleiden, die Toilettenbenutzung, den Bett‑/Rollstuhltransfer und die Kontinenz umfassen, wurde von Katz et al. [24] 1963 ein geriatrisches Assessment entwickelt, das heute noch eingesetzt wird. Funktionsverluste durch das Altern, Krankheit und Behinderung können allerdings dazu führen, dass die Selbstpflegeaktivitäten des täglichen Lebens nicht oder nur noch sehr eingeschränkt wahrgenommen werden können. Diese Personen benötigen bei gravierenden Funktionsverlusten Unterstützung in der Bewältigung des Alltags. Im Jahr 2019 gab es 7,9 Mio. schwer behinderte Menschen – also Menschen, denen von den Versorgungsämtern ein Grad der Behinderung (GdB) von 50 und mehr zuerkannt wurde – in Deutschland; davon waren mehr als 78 % 55 Jahre und älter [10]. Das Statistische Bundesamt veröffentlicht alle 2 Jahre eine detaillierte Auswertung u. a. zu der Art und dem Umfang der Behinderungen [26]. Einen Überblick über die Zahl sowie den GdB der Personen, die als erste Behinderung schwerste Einschränkungen der Arme haben und somit auch beim Essen und beim Trinken stark eingeschränkt sind, gibt Tab. 1.

**Table 1:** 

Art der schwersten Behinderung	Grad der Behinderung							
	Insgesamt	50	60	70	80	90	100	80–100
**Verlust/Teilverlust**								
– Beide Arme	1768	495	221	156	148	89	659	896
– 3 oder 4 Gliedmaßen	1202	266	138	130	152	102	414	668
**Funktionseinschränkungen**								
– Beide Arme	22.755	11.298	4376	2465	1771	730	2155	4616
– Beide Arme und Beine	82.405	24.056	13.774	10.675	10.015	4978	18.907	33.900
Querschnittlähmung	16.202	226	228	383	817	465	14.083	15.365
**Gesamt**	**124.332**	**36.341**	**18.737**	**13.809**	**12.903**	**6364**	**36.178**	**55.445**

Diese Zahlen geben einen Hinweis darauf, wie viele Menschen eine Assistenz für die Nahrungsaufnahme benötigen. Mehr als 124.000 Menschen haben (Funktions‑)Verluste der Arme oder eine Querschnittlähmung. Davon haben 55 % nicht nur eine, sondern eine weitere oder mehrere weitere Behinderungen.

Dazu kommen mehr als 620.000 Menschen, die neben ihrer schwersten Behinderung auch (Teil‑)Verluste oder Funktionseinschränkungen ihrer Gliedmaßen haben [26]. Da die weiteren Behinderungsarten lediglich mit der Oberkategorie präsentiert werden, werden in der Kategorie „Querschnittlähmung, zerebrale Störungen, geistig-seelische Behinderungen, Suchtkrankheiten“ 574.301 Personen mit einer zweiten oder weiteren Behinderung aufgeführt [26].

## Zielsetzung

Ziel dieses Beitrags ist es, einen exemplarischen Überblick zu geben, welche robotischen Systeme als Hilfsmittel schon heute für die Basisaktivität Nahrungsaufnahme zur Verfügung stehen. Dank des technologischen Fortschritts sind mittlerweile verschiedene Produkte auf dem Markt verfügbar. Da sich die Funktionseinschränkungen der betroffenen Personen in der Regel sehr unterscheiden, gibt es keine „One-fits-all“-Technologien und eine Reihe von Herausforderungen im Versorgungsprozess.

## Methodik

Die vorgestellten Ergebnisse basieren auf Literatur- und Produktrecherchen im Rahmen verschiedener Forschungsprojekte (I-Supported Bath Systems [18]; CareV.E.T. [4]; Studie Robotik in der Gesundheitswirtschaft [19], MobILe [21]) sowie Forschungsaufenthalten der Erstautorin an der Osaka-Universität. Des Weiteren wurden die Internetplattformen von „rehadat-hilfsmittel.de“ und des GKV-Spitzenverbandes „https://hilfsmittel.gkv-spitzenverband.de/home.action“ für die Recherchen herangezogen.

## Basisaktivitäten: Essen und Trinken

Essen und Trinken sind zentrale Bestandteile des Lebens. Wenn Arm- und Handfunktionen eingeschränkt sind, existieren in Gewicht, Form- und Farbgebung angepasste Ess- und Trinkhilfen wie Besteckhalter, Griffverdickungen und -verlängerungen für Bestecke, Halterungen für Becher, Tellerranderhöhungen oder Saug- und Trinkhilfen, die bei Restgreiffunktionen, die selbstständige Nahrungs- und Getränkeaufnahme unterstützen. Das Portal rehadat-hilfsmittel.de listet in seiner Rubrik „Haushalt und Ernährung“ im Bereich „Essen und Trinken“ mehr als 160 Produkte dazu auf [13].

Sind die Funktionsverluste umfassender, sodass die Arme und Hände nicht mehr genutzt werden können, stehen Essapparate zur Verfügung. Diese Essapparate sind meist Bestecke wie Löffel oder Gabel, die an einen Gelenkarm montiert sind, der wiederum an einem Teller befestigt ist. Diese können mit einzelnen Fingern, Arm, Knopfdruck oder auch mithilfe eines Fußschalters bedient werden.

Rehadat-hilfsmittel.de führt 4 Systeme auf [14]: Neater Eater der Fa. Neater Solutions, Buxton, Großbritannien, iEat-Essroboter der Fa. Assistive Innovations BV, Didam, Niederlande sowie die Esshilfen Nelson und Obi der Fa. Focal Meditech BV, Tilburg, Niederlande. Darüber hinaus gibt es die Esshilfe bestic der Fa. Camanio Care AB, Stockholm, Schweden [9]. Mit diesen Essapparaten sollen informell und formell Pflegende entlastet werden. Die betroffenen Personen können eigenständig essen. Mit dem Gerät können sie zudem selbst bestimmen, was, in welcher Reihenfolge und in welchem Tempo sie essen. Dadurch sollen der Genuss beim Essen und die Lebensqualität gefördert werden [1, 22, 25, 27, 28]. Eine qualitative Studie, an der sich 23 Personen mit neuromuskulären Erkrankungen beteiligten, die seit mindestens 3 Monaten einen Neater Eater nutzten, zeigte eine Reihe positiver Effekte auf das Selbstwertgefühl und die Selbstständigkeit, aber auch, dass die Einstellung zu dem Gerät eine Rolle spielte [20].

Darüber hinaus sind auf rehadat-hilfsmittel.de 3 Roboterarme aufgeführt, die komplexe Greif- und Haltefunktionen übernehmen und damit auch die Nahrungsaufnahme unterstützen können: iARM (Fa. Assistive Innovations BV, Didam, Niederlande, [15]), Kinova JACO Roboterarm (Fa. Kinova Inc., Boisbriand, Kanada, [16]) und BATEO (Fa. EXXOMOVE, Bayreuth, Deutschland, [17]). Diese Systeme richten sich an Menschen mit geringer/fehlender Muskelfunktion in Händen und Armen, amyotrophischer Lateralsklerose (ALS), Querschnittlähmung etc. Die robotischen Arme werden am Elektrorollstuhl befestigt und können neben der Nahrungsaufnahme für vielfältige Aufgaben genutzt werden. So berichtete eine Teilnehmerin einer Fokusgruppe des BMBF-Projektes MobILe [21], dass sie sich damit schminken und selbst kratzen, Türen öffnen und bei der Essenszubereitung mitwirken könne. Bedient werden die Roboterarme über einen Joystick, ein Tastenfeld oder eine individuell angepasste Sondersteuerung. Da es Personen gibt, die die genannten Interfaces nicht bedienen können, wird z. B. im Rahmen des BMBF-Projektes MobILe [21] an einer Robotersteuerung über Kopf- und Augenbewegungen gearbeitet, sodass mit dem Roboterarm über ein Headset mit Bewegungssensoren oder einer Brille mithilfe eines Eyetracker und der Elektrookulographie interagiert werden kann. Eine qualitative Untersuchung von 7 Personen, die einen JACO-Arm mindestens ein halbes Jahr benutzten, kommt zu überwiegend positiven Ergebnissen, da sie damit Alltagstätigkeiten ausführen können, die sonst nicht möglich wären. Die Nutzung von JACO wirkte sich positiv auf das psychosoziale Befinden aus [2].

## Ausgewählte hemmende und fördernde Faktoren des Versorgungsprozesses

### Information und Qualifizierung

Viele Informationen über assistive Technologien, Hilfsmittel und Robotik sind im Internet verfügbar. In Deutschland können Informationen rund um Hilfsmittel und assistive Technologien (Oberbegriff der WHO) auf der Internetplattform www.rehadat-hilfsmittel.de abgerufen werden. Diese Plattform wird vom Institut der deutschen Wirtschaft Köln e. V. betrieben sowie vom Bundesministerium für Arbeit und Soziales (BMAS) und aus dem Ausgleichsfonds gefördert. Sie ist eine profunde Quelle und hat das Ziel, herstellerneutral zu Hilfsmitteln und technischen Arbeitshilfen in Deutschland zu informieren [12]. Hier werden auch Produkte aufgenommen, die nicht im Hilfsmittelverzeichnis des GKV-Spitzenverbands gelistet sind [8]. Die rund 32.000 Produkte im GKV-Hilfsmittelverzeichnis sind in 41 Produktgruppen mit rund 800 Produktuntergruppen sowie ca. 2600 Produktarten gegliedert [6]. Ärzte/Ärztinnen finden hier die für die Verordnung relevanten Positions- und Produktnummern, die eine Kostenübernahme des jeweiligen Leistungsträgers vereinfacht – es sei denn, dass es sich um Gebrauchsgegenstände des täglichen Lebens handelt [7].

Trotzdem ist das Wissen in Bezug auf Hilfsmittel und für die Unterstützung der Nahrungsaufnahme weder bei den betroffenen Menschen noch in den Gesundheitsberufen in ausreichendem Maß verbreitet. Dazu kommt der schnelle technologische Wandel mit einer Vielzahl von Neuentwicklungen. Die Veränderungen spiegelt beispielsweise das Update des GKV-Hilfsmittelverzeichnisses [7] mit 365 neu eingestellten Produkten und Änderungen bei weiteren 370 Produkten wider. Hilfsmittel, assistive Technologien und digitale Gesundheitsanwendungen sind kein obligatorischer Part der medizinischen Ausbildung, obwohl Ärzte/Ärztinnen für die Verordnung von Hilfsmitteln zuständig sind. Deshalb sind diese auf gute Kollaborationsstrukturen mit den anderen Gesundheitsberufen wie Pflege, Ergotherapie, Physiotherapie oder Logopädie angewiesen. Doch auch hier sind Hilfsmittel oder assistive Technologien weder systematisch in Aus‑, Fort- und Weiterbildung integriert, noch werden ausreichende Fortbildungsmöglichkeiten angeboten, wie eine im Rahmen des EU-Projekts DDSkills durchgeführte Recherche ergab [5]. Lediglich die „traditionellen“ Hilfsmittel sind Teil der therapeutischen Ausbildung, v. a. in der Ergotherapie.

Betroffene Personen und ihre Angehörigen müssen sich gleichfalls durch einen Dschungel an Informationen durcharbeiten, bis sie auf passende Produkte stoßen – gerade in kritischen Lebenssituationen und/oder bei fehlendem Internetzugang/fehlenden Internetkenntnissen ist dies schier nicht zu bewältigen.

Wünschenswert und notwendig sind – aus der Perspektive der Autorinnen – Informationen quasi „auf Knopfdruck“ für die Betroffenen und Transparenz zu den Versorgungsabläufen. Zum anderen werden geeignete Qualifizierungsangebote für die verschiedenen Berufsgruppen benötigt, um einen „best fit“ von Funktionseinschränkungen und Hilfsmitteln zu ermöglichen. Nicht geklärt ist, welche Akteure dieses gerade bei technisch hochkomplexen Produkten leisten können. Heute sind für das „Fitmachen“ in der Technologie die Leistungserbringer wie z. B. Sanitätshäuser in der Verpflichtung. Hier stellt sich die Frage, ob es neben dem betreuenden Hersteller/Vertrieb eine neue Berufsgruppe geben muss, die in der Lage ist, sich um Wartung, Reparaturen und Sicherstellung des laufenden Betriebes komplexer Technologien und der Einweisung in die Nutzung zu kümmern. Diese Gesichtspunkte sind auch bei der Entwicklung solcher Systeme in Betracht zu ziehen, um eine hohe Gebrauchstauglichkeit und Systemsicherheit zu erreichen.

### Kostenerstattung durch die gesetzlichen Krankenversicherungen

Versicherte haben nach SGB V, § 33 Hilfsmittel, Abs. 1 [23] „Anspruch auf Versorgung mit … Hilfsmitteln, die im Einzelfall erforderlich sind, um den Erfolg der Krankenbehandlung zu sichern, einer drohenden Behinderung vorzubeugen oder eine Behinderung auszugleichen, soweit die Hilfsmittel nicht als allgemeine Gebrauchsgegenstände des täglichen Lebens anzusehen …sind“. Ess- und Trinkgeschirr sind Gebrauchsgegenstände des täglichen Lebens und unterliegen damit nicht der Leistungspflicht. Behindertengerechte Anpassungen können leistungspflichtig sein oder im Ermessen der jeweiligen Krankenkasse liegen.

Im GKV-Hilfsmittelverzeichnis findet sich unter den leistungsrechtlichen Hinweisen, dass die Versorgungsnotwendigkeit anhand der Gegebenheiten des Einzelfalls bewertet werden muss. Erforderlich sind eine Gesamtbetrachtung der individuellen Situation, Bedarfsklärung mit der Person unter Einbindung eines/einer Ergotherapeuten/Ergotherapeutin bzw. Logopäden/Logopädin und eine ausführliche ärztliche Begründung [11].

Von den im Portal rehadat-hilfsmittel.de aufgeführten, technisch komplexeren Essapparaten ist lediglich dem Neater Eater in der mechanischen Variante eine Hilfsmittelnummer zugeordnet. Eines der robotischen Produkte hat eine Hilfsmittelnummer beantragt; bei den anderen wird auf die leistungsrechtlich möglichen Einzelfallentscheidungen verwiesen, die eine detaillierte Begründung benötigen. Hier ist das Zusammenspiel der Gesundheitsberufe gefragt, um eine zügige(re) Hilfsmittelversorgung der Betroffenen zu erreichen, denn die Praxis zeigt, dass eine Verordnung oft abgelehnt wird und dann in den Widerspruch gegangen werden muss. Zeitspannen bis zu einer Bewilligung können bis zu 2 Jahre und länger dauern, eine Zeit, die für die betroffenen Menschen sehr belastend ist und in der sie auch versterben können. Die Beantragung von innovativen Hilfsmitteln ist von einem komplexen administrativen Prozess begleitet. Kostenintensive Produkte werden häufig abgelehnt. Mögliche Widerspruchsverfahren bedeuten für die Betroffenen hohe Belastungen. Fehlen das Wissen oder die Energie, diese Ansprüche durchzusetzen, kann dies die Lebensführung, Autonomie und Teilhabemöglichkeiten erheblich einschränken.

Diese Versorgungsproblematik wird auch für andere Hilfsmittel, beispielsweise von Besuchern/Besucherinnen der Ausstellung „Barrierefreies Wohnen und Leben“ der Frankfurt University of Applied Sciences oder auch in der Literatur, berichtet [3, 29]. Hier besteht Forschungsbedarf zum Ausmaß der Ablehnungen, zu den durchgeführten Widersprüchen sowie den Auswirkungen auf die Effekte und Versorgung der Betroffenen.

## Fazit für die Praxis

- Für Menschen mit Funktionseinschränkungen und -verlust der selbstständigen Nahrungsaufnahme existiert eine Reihe von Hilfsmitteln und robotischen Produkten. Mit diesen Essapparaten sollen informell und formell Pflegende entlastet werden.
- Studienergebnisse zeigten positive Effekte auf das Selbstwertgefühl, die Selbstständigkeit, das psychosoziale Befinden und die Lebensqualität der Betroffenen, aber auch, dass die Einstellung zu dem Gerät eine Rolle spielt.
- Das Wissen bezüglich entsprechender Hilfsmittel ist bislang weder bei den betroffenen Menschen noch in den Gesundheitsberufen in ausreichendem Maß verbreitet.
- Ess- und Trinkgeschirr unterliegen i. Allg. nicht der Leistungspflicht. Behindertengerechte Anpassungen können leistungspflichtig sein oder im Ermessen der jeweiligen Krankenkasse liegen. Hier ist das Zusammenspiel der Gesundheitsberufe gefragt, um eine zügige(re) Hilfsmittelversorgung der Betroffenen zu erreichen.
- Mögliche Widerspruchsverfahren bedeuten für die Betroffenen hohe Belastungen. Fehlen das Wissen oder die Energie, die Ansprüche durchzusetzen, kann dies die Lebensführung, Autonomie und Teilhabemöglichkeiten erheblich einschränken.

## References

[1] Al-Halimi R, Moussa M (2017). Performing complex tasks by users with upper-extremity disabilities using a 6-DOF robotic arm: a study. IEEE Trans Neural Syst Rehabil Eng.

[2] Beaudoin M, Lettre J, Routhier F, Archambault PS, Lemay M, Gélinas I (2019). Long-term use of the JACO robotic arm: a case series. Disabil Rehabil Assist Technol ..

[3] Blomenkamp L, von Kamen R, Lagedroste C, Seifen S (2014). Probleme der Hilfsmittelversorgung im Übergang aus dem Krankenhaus in die häusliche Pflegesituation. Evaluationsbericht.

[4] Care VET (2020) Entwicklung von Qualifizierungsmaßnahmen zu Sensorik für Beschäftigte im Sozialwesen. Erasmus+. Strategic Partnership (016-1-EL01-KA202-023612. 2016–2018). www.carevet.eu. Zugegriffen: 17. Aug. 2020

[5] DDSkills—Cutting-Edge Digital Skills for professional care givers of Persons with Disabilities and mental health Problems (Erasmus+ Strategic Partnership. 612655-EPP-1-2019-1-EL-EPPKA2-SSA; 2020–2022) www.ddskills.eu

[6] GKV-Spitzenverband (2019). 2. Bericht des GKV-Spitzenverbandes zur Fortschreibung des Hilfsmittelverzeichnisses gemäß § 139 Absatz 9 Satz 3 SGB VBBGK Berliner Botschaft.

[7] GKV-Spitzenverband (2020). 3. Bericht des GKV-Spitzenverbandes gemäß $139 Absatz 9 Satz 3 SGB zur Fortschreibung des Hilfsmittelverzeichnisses. Berichtsraum: 01.01.2019–29.02.2020.

[8] https://hilfsmittel.gkv-spitzenverband.de/home.action Zugegriffen: 05. August 2020

[9] https://www.camanio.com/us/products/bestic/. Zugegriffen: 04. August 2020

[10] https://www.destatis.de/DE/Presse/Pressemitteilungen/2020/06/PD20_230_227.html

[11] https://www.rehadat-gkv.de/info/index.html?pgnr=2&pginfo=true Änderungsdatum 13. Nov. 2018. Zugegriffen: 04. August 2020

[12] https://www.rehadat-hilfsmittel.de/de/informationen/ueber-uns/ Zugegriffen: 05. August 2020

[13] https://www.rehadat-hilfsmittel.de/de/produkte/haushalt-ernaehrung/ess-und-trinkhilfen/ Zugegriffen: 17. August 2020

[14] https://www.rehadat-hilfsmittel.de/de/produkte/haushalt-ernaehrung/ess-und-trinkhilfen/essapparate/. Zugegriffen: 16. August 2020

[15] https://www.rehadat-hilfsmittel.de/de/produkte/haushalt-ernaehrung/greifen-tragen-halten/greifvorrichtungen/index.html?reloaded&sort=score+desc&page=6&mode=detail. Zugegriffen: 16. August 2020

[16] https://www.rehadat-hilfsmittel.de/de/produkte/haushalt-ernaehrung/greifen-tragen-halten/greifvorrichtungen/index.html?reloaded&sort=score+desc&page=7&mode=detail. Zugegriffen: 16. August 2020

[17] https://www.rehadat-hilfsmittel.de/de/produkte/haushalt-ernaehrung/greifen-tragen-halten/greifvorrichtungen/index.html?reloaded&sort=score+desc&page=5&mode=detail. Zugegriffen: 16. August 2020

[18] I‑Supported Bath Robots – Entwicklung eines robotischen Duschsystems. Akzeptanz, Usability und Ethik. (EU: HORIZON 2020 EU:643666. 2015–2018) http://www.i-support-project.eu/. Zugegriffen: 17. August 2020

[19] Klein B, Graf B, Schlömer I, Roßberg H, Röhricht K, Baumgarten S (2018). Robotik in der Gesundheitswirtschaft. Einsatzfelder und Potenziale.

[20] Mandy A, Sims T, Stew G, Onions D (2018). Manual feeding device experiences of people with a neurodisability. Am J Occup Ther.

[21] MobILe (2020) Physische Mensch-Roboter-Interaktion für ein selbstbestimmtes Leben (BMBF, Fördernr.: 16SV7868, 2017–2020). https://www.technik-zum-menschen-bringen.de/projekte/mobile. Zugegriffen: 5. Aug. 2020

[22] Naotunna I (2015). Meal assistance robots: A review on current status, challenges and future directions. 2015 IEEE/SICE International Symposium on System Integration (SII).

[23] SGB V, § 33, 1 (https://www.gesetze-im-internet.de/sgb_5/__33.html Zugegriffen: 06. August 2020)

[24] Shelkey M, Wallace M (2012). Katz index of independence in activities of daily living. try this; best practices in nursing care to older adults. Issue no 2.

[25] Song WK, Bien Z, Zenn, al (2010). Design of novel feeding robot for Korean food. Aging friendly technology for health and independence.

[26] Statistisches Bundesamt (2019). Sozialleistungen Schwerbehinderte Menschen 2017.

[27] Thinh NT, Thang LH, Thanh TT (2017). Design strategies to improve self-feeding device-FeedBot for Parkinson patients. International Conference on System Science and Engineering (ICSSE).

[28] Thinh NT, Tho TP, Tan NT (2017). Designing self-feeding system for increasing indep endence of elders and Parkinson people. 17th International Conference on Control, Automation and Systems (ICCAS).

[29] Dierks C, Retter S, Pirk J, Verbraucherzentrale Bundesverband e. V (2019). Rechtsgutachten. Möglichkeiten der Kostenerstattung technischer Assistenzsysteme (AAL) für pflegebedürftige Verbraucherinnen und Verbraucher nach geltendem Recht sowie Entwicklung von konkreten Handlungsempfehlungen.

